# Eco-Friendly Inorganic Binders: A Key Alternative for Reducing Harmful Emissions in Molding and Core-Making Technologies

**DOI:** 10.3390/ijms25105496

**Published:** 2024-05-17

**Authors:** Angelika Kmita, Rafał Dańko, Mariusz Holtzer, Józef Dańko, Dariusz Drożyński, Mateusz Skrzyński, Agnieszka Roczniak, Daniel Robert Gruszka, Jarosław Jakubski, Sara Tapola

**Affiliations:** 1 Academic Centre for Materials and Nanotechnology, AGH University of Krakow, A. Mickiewicza 30 St., 30-059 Krakow, Poland; 2 Faculty of Foundry Engineering, AGH University of Krakow, A. Mickiewicza 30 St., 30-059 Krakow, Poland; rd@agh.edu.pl (R.D.); holtzer@agh.edu.pl (M.H.); jd@agh.edu.pl (J.D.); dd@agh.edu.pl (D.D.); mskrzyns@agh.edu.pl (M.S.); arocznia@agh.edu.pl (A.R.); dgruszka@agh.edu.pl (D.R.G.); jakubski@agh.edu.pl (J.J.); 3 Meehanite Technology Oy, Kuokkamaantie 4, 33800 Tampere, Finland; sara.tapola@meehanite.fi

**Keywords:** reducing harmful emissions, inorganic binders, green casting, sustainable polymeric materials

## Abstract

Many years of foundry practice and much more accurate analytical methods have shown that sands with organic binders, in addition to their many technological advantages, pose risks associated with the emission of many compounds, including harmful ones (e.g., formaldehyde, phenol, benzene, polycyclic aromatic hydrocarbons, and sulfur), arising during the pouring of liquid casting alloys into molds, their cooling, and knock-out. The aim of this research is to demonstrate the potential benefits of adopting inorganic binders in European iron foundries. This will improve the environmental and working conditions by introducing cleaner and more ecological production methods, while also ranking the tested binders studied in terms of their harmful content. The article pays special attention to the analysis of seven innovative inorganic binders and one organic binder, acting as a reference for emissions of gases from the BTEX (benzene, toluene, ethylbenzene, and xylenes) and PAHs (polycyclic aromatic hydrocarbons) groups and other compounds such as phenol, formaldehyde, and isocyanates (MDI and TDI) generated during the mold pouring process with liquid metals. The knowledge gained will, for the first time, enrich the database needed to update the Reference Document on The Best Available Techniques for the Smitheries and Foundries Industry (SF BREF).

## 1. Introduction

### 1.1. General Eco-Technological Characteristics of Currently Used Molding and Core Sands

For many years, sand casting technology was dominated by sands containing bentonite (green sand) and sands bound with organic binders, in which various forms of resins and hardeners (catalysts) were used as binders. Sands with inorganic binders were much less numerous, represented mainly by molding sands bound with water glass [1,2,3]. The introduction of water glass as a binder in the early years of the 20th century played a very important role in increasing the efficiency of mold and core production [1,4]. Processes using water glass bonded molding sands cured with CO_2_ or liquid organic hardeners helped to improve the quality of the working and natural environment. However, these sands had some technological disadvantages (poor knock-out, difficult regeneration, and strong alkalization of wastes), and their use was declining; hence, work was undertaken on new generations of molding sands [2,5]. 

In 2020, the foundry industry in the EU employed more than 240,000 people [6]. Additionally, foundries play a key role in other industries, supplying various cast components to the automotive, shipbuilding, cosmic, and construction sectors, among many other key industries. Despite the continuous development of green technologies, work is still underway to reduce the negative impact of the foundry on the environment [1,7,8,9], with particular emphasis on air quality [10,11,12,13].

In this hereby work, the quality assessment of molding sands used the aspect of their harmfulness as a priority criterion, or at least as the equivalent to the assessment of the expected and achievable technological properties of the molding sand, suitable for obtaining a good casting. For this reason, the current system of domination and projection of environmental conditions [14,15,16,17,18] onto casting technology can be conventionally referred to by the acronym FET (Feasible Ecological Technology). 

Up to now, the subject of numerous investigations has been organic binders, while inorganic binders have been studied to a much lower degree [5,19,20] because only their harmfulness was tested. As everybody knows, molding sands in contact with liquid foundry alloys generate several dangerous substances [1,19,21]. Moreover, studies presented in papers [20,22] have indicated that molding sands with bentonite can also release dangerous substances from the PAH and BTEX groups since they contain carbon compounds. That is why, taking into account the high prices of binders and their negative influence on the environment, studies concerning the application of inorganic binders instead of organic ones started in a wider than thus far scope.

### 1.2. Mold Sands with Organic Binders

**Figure 1:**
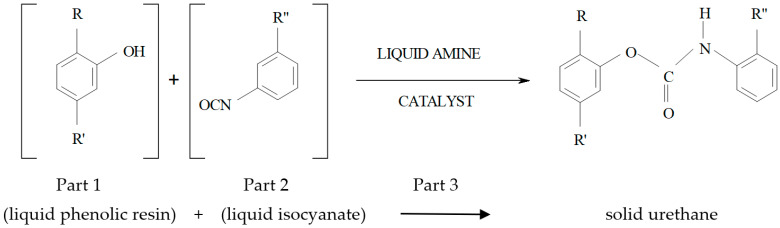
Chemical reaction between binder components in the PUNB organic binder system.

The result of research studies [2,23] constituted new or modified binders (used previously). An example of such binders is the polyurethane binder of the “no bake” system used in the Phenolic Urethane No Bake (PUNB) technology [24,25]. The PUNB technology allows for the high efficiency of castings of very good quality. This mold sand is characterized by high strength, so that the addition of a binder can be reduced to a minimum and important environmental benefits (e.g., less hazardous ingredients, low odor and low smoke, reduction in phenol pollution levels, and improved community relations and work environment) as well as technological and economic benefits (such as fast strip time and good through-cure, good strength of new and mechanically reclaimed sand, high reuse percentage for reclaimed sand, very high productivity, good casting quality, and lower landfill costs) [1,4,26]. The bonding process mechanism involves a polyaddition reaction between a phenolic resin (polyol component) and isocyanate components (Figure 1). The PUNB process is made of three parts, such as phenol formaldehyde resin, considered Part 1; Part 2 is a polymeric MDI-type isocyanate, and an amine catalyst is Part 3. The curing mechanism of the PUNB system is shown in Figure 1 [25,27].

**Figure 2:**
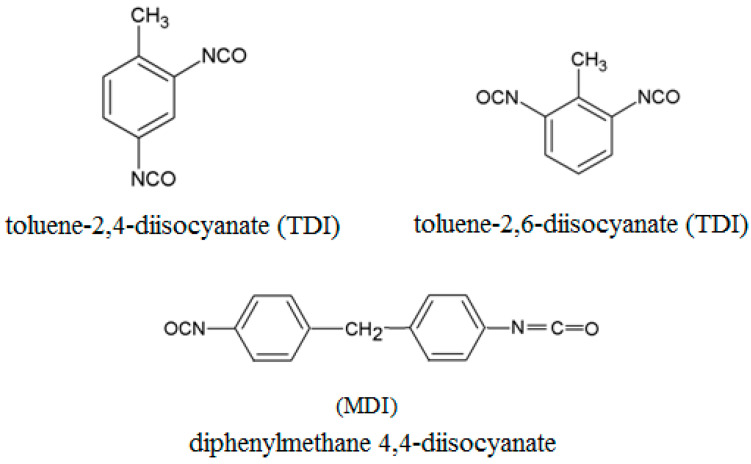
Examples of the most often applied aromatic isocyanates [1].

The binder level required for this system is 0.7 to 2% by the weight of sand. Part 2 is in terms of 40–45% on the basis of Part 1. The third part catalyst level is also decided by the weight of Part 1, and it is 0.4 to 8%. In a chemical reaction between Part 1 and Part 2 (Figure 1), it forms a urethane bond only (without other by-products) [27]. Depending on the raw material composition and reaction conditions, polyurethanes can contain either polyethers or polyesters, in which hydroxy groups are present at the ends of the chain [28]. The most broadly applied are 4,4′-diphenylmethane diisocyanate (MDI) and toluene-2,4-diisocyanate (TDI) (Figure 2).

The generally positive assessment of PUNB sands in the foundry environment clashes with the increasingly stringent environmental regulations and growing public awareness, as well as with access to increasingly accurate analytical methods that radically narrow the limits of harmfulness assessment.

### 1.3. Mold Sands with Inorganic Binders

The significantly limited scope for improving organic binders improvements, especially in terms of dramatically reducing negative environmental impacts, has led to a resurgence in research into inorganic binders. This is particularly relevant for cores, which typically constitute up to 10% of the mold volume but emit up to 70% of the volatile organic compounds (VOCs) in a foundry. These compounds are a wide group of organic pollutants that are currently receiving a lot of attention in environmental science. The main representatives of VOCs are benzene, toluene, ethylbenzene, and xylene (BTEX group) [10,29]. The main advantage of molding sands with inorganic binders (e.g., water glass with sodium or potassium silicate) is their low price and low harmfulness.

The following inorganic binders are based on silicate or alumina–silicate compounds: CORDIS (Huttenes–Albertus, Hannover, Germany), alternative warm box binder (AWB–Nemak, Dillingen, Germany), INOTEC (ASK Chemicals Austria GmbH, Vienna, Austria), SOLOSIL, AMASILIK (FOSECO, Tamworth, UK), GEOPOL (SAND TEAM, Holubice, Czech Republic) [30], etc. 

On the other hand, the highest defect of so far applied systems with, e.g., water glass, is their bad knocking-out and difficult regeneration. Molding sands with silicate binders are characterized by low strength in the first phase of hardening, when their hardening process is not complete. The consequence of this is an increase in strength in parts of the mold further away from the casting, which is the cause of problems with the knock-out properties of the molding sand with this binder. The durability of molds and cores of molding sands with water glass is limited due to the fact that they become brittle and hygroscopic after hardening. Moreover, they show low susceptibility to mechanical regeneration, which is virtually the only effective method for dry recovery of the quartz matrix [1].

All these constitute the reason that, from a technological point of view, these sands cannot be widely applied in cast iron or cast steel foundry practices [1]. That is why the producers of binding materials, in order to improve the quality of the offered inorganic binders from the silicate or geopolymer groups, are still refining their knock-out properties as well as their reclamation abilities. They apply several treatments, e.g., modifications of the organic binding structure or various additions, which can improve the technological properties of sands. 

The subject and aim of this study was the determination of the gas forming tendency and composition of gases emitted by new, innovative inorganic binders with respect to environmental protection [31].

## 2. Results and Discussion

### 2.1. Volume of Emitted Gases

**Figure 3:**
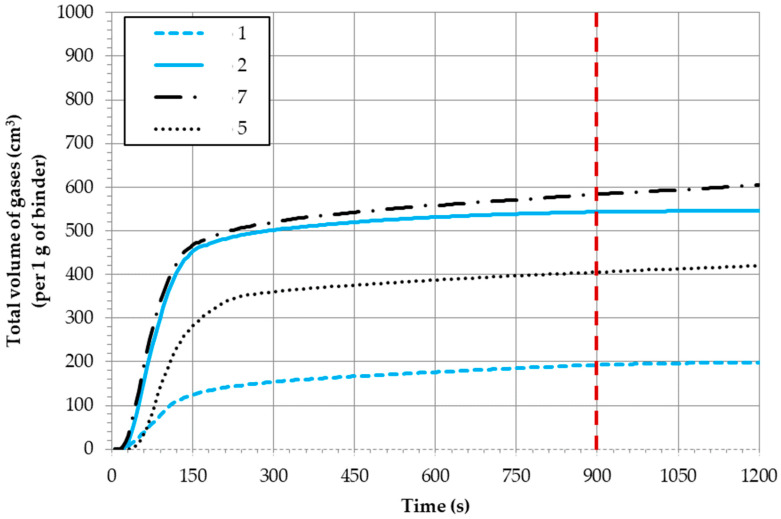
Emissivity of gases from the investigated molding sands and influence of the hardening method (red dashed line—the contractual time for completing the examined process).

**Figure 4:**
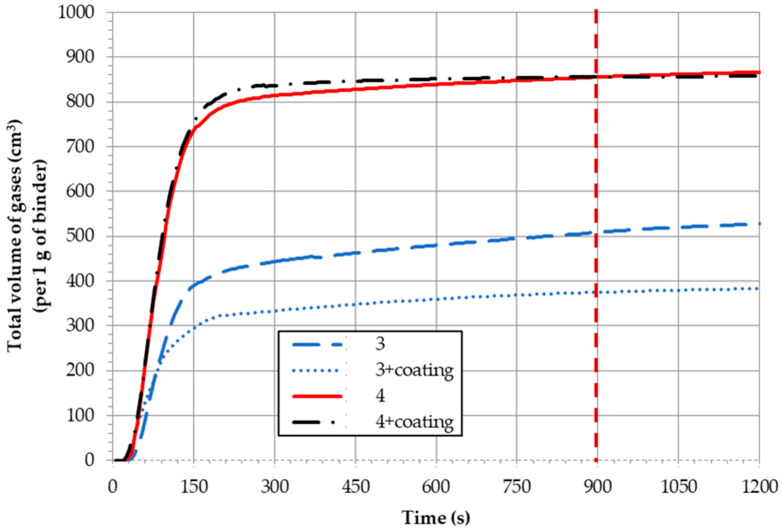
Emissivity of gases from the investigated molding sands and influence of the protective coating (red dashed line—the contractual time for completing the examined process).

**Figure 5:**
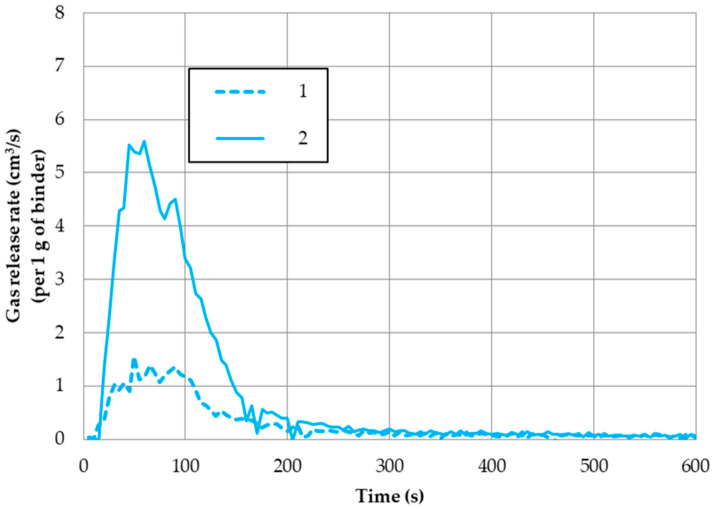
Gas release rate from the investigated molding sands: influence of the applied hardening technology.

The following figures (Figure 3 and Figure 4) illustrate selected measurement results of the total volume of gases and the gas released rates (Figure 5), calculated per 1 g of a binder from the investigated molding sands. Table 1 summarizes the data regarding the amount of gases released from all tested systems.

Based on the research results—which are possible to obtain—it can be concluded that the mold sand bonded by an organic binder (code no. 4, which is the reference sand) released the largest volume of gases during pouring (V_900_–855 cm^3^ for 1 g of binder) (see Table 1). The smallest amount of gases during pouring was emitted by mold sand code no. 1 (bonded by a geopolymer binder hardened by means of the warm box method), for which V_900_ was 192 cm^3^ per 1 g of a binder, while the highest volume of gases (out of sands with an inorganic binder) was released by sand no. 7 (V_900_ was 583.97 cm^3^ per 1 g of a binder). The amount of gases emitted from samples of molding sands bonded by a geopolymer binder depended on the hardening method (Figure 3 and Table 1). The sample hardened by CO_2_ emitted much more gases than the sand hardened by the warm box technology (Table 1). Sands bonded by the binder in the form of a silicate mixture (code no. 5 and code no. 7) behaved in a similar way. These sands that were hardened by CO_2_ also had a higher emission of gases in relation to sands that were hardened by the warm box method (Figure 3 and Table 1).

Measurements of sands of codes no. 3 and no. 4 were also performed with the application of water-based protective coating type A on them (Figure 4). In cases of sand bonded by an organic binder (code no. 4), the application of a water-protective coating was not performed, resulting in differences in the volume of emitted gases (code no. 4 + coating). These sands—out of all the tested sands—had the highest emission rates of gases as well as the highest volume values of released gases (Figure 4 and Table 1). However, in cases where the sand was bonded by an inorganic binder (code no. 3), the presence of coating (code no. 3 + coating) caused an approximately 50% decrease in the amount of emitted gases (Figure 4 and Table 1).

In practice, the emission rate of all of the investigated sands was at its maximum after approximately 60 s of mold pouring (Table 1). The curves of gases emission rate in dependence of the hardening way of sands (code no. 1 and code no. 2) are presented in Figure 5. It can be seen that in the case of sand code no. 1 (hardened in the warm box technology), gases are emitted slowly, not in a rapid way, and their amount is four-times lower than from the reference sand (code no. 4) (Table 1). This smaller amount of gases emitted at a low rate, especially in the first seconds after pouring, is essential to the value of the surface quality of the produced castings. 

### 2.2. Emission of Harmful Gases

During the casting production, as a result of the influence of high temperatures of the casting alloy in contact with molding sand, the thermal decomposition of organic components of the sand occurs. In effect, several harmful compounds, both gaseous and dust, are formed. Among the gaseous products being formed, two groups of substances of carcinogenic and mutagenic character, which are especially dangerous for employees, should be pointed out. Also, substances from the BTEX group (benzene, toluene, ethylbenzene, and xylenes), PAHs group (polycyclic aromatic hydrocarbons), and greenhouse gases: CO, CO_2_, NO_x_, SO_2_, and T_voc_ are very dangerous.

#### 2.2.1. BTEX Emissions

**Figure 6:**
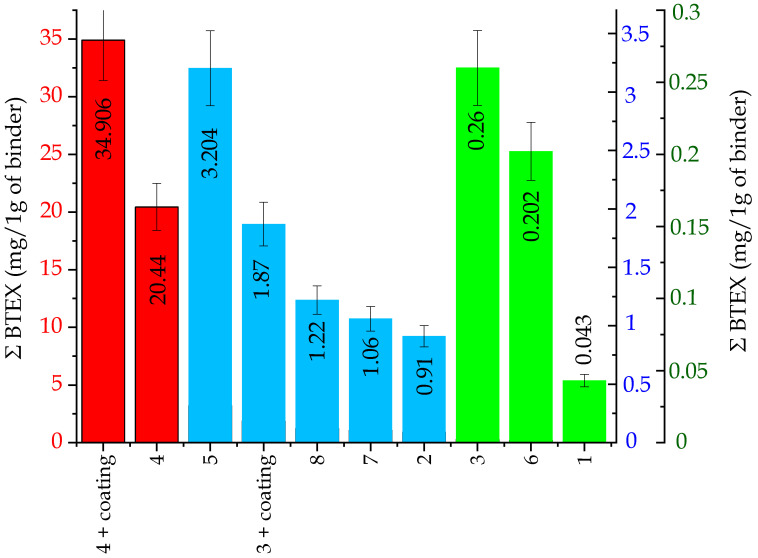
Total emission of substances from the BTEX group, calculated per 1 g of a binder (I red group (code: no. 4, no. 4 + coating)—very high content of compounds from the BTEX group; II blue group (code: no. 3 + coating, 5, 8, 7, 2)—small content of compounds from the BTEX group; III green group (code: no. 3, 6, 1)—trace content of compounds from the BTEX group).

The total emissions of substances from the BTEX group, released by individual tested molding sands (per 1 g of a binder), are presented in Figure 6, while in Table 2, the contents of the substances from this group are listed. As can be seen, it is possible to single out three groups of binders that differ considerably in amounts of released substances from the BTEX group in emitted gases (Figure 6):

- I red group, code: no. 4, no. 4 + coating—very high content of compounds from the BTEX group.
- II blue group, code: no. 3 + coating, 5, 8, 7, 2—small content of compounds from the BTEX group.
- III green group, code: no. 3, 6, 1—trace content of compounds from the BTEX group.

**Table 2:** Contents of individual substances from the BTEX group generated during the thermal decomposition of molding sands.

BTEX	Emission of Individual Substances (mg/per 1 g of a Binder)									
	Molding Sands Code									
	1	2	3	3 + Coating	4	4 + Coating	5	6	7	8
Benzene	0.042	0.881	0.256	1.793	19.338	32.286	3.163	0.199	1.038	1.203
Toluene	0.001	0.017	0.003	0.004	0.952	0.514	0.041	0.002	0.021	0.000
Ethylbenzene	<	0.010	0.002	0.073	0.003	1.574	<	0.001	0.002	0.016
Xylenes	<	0.003	0.000	0.007	0.154	0.532	<	0.000	0.002	0.000
Σ BTEX	0.043	0.912	0.261	1.877	20.446	34.906	3.204	0.202	1.064	1.220
the value of +/− 10% on account	0.004	0.091	0.026	0.188	2.045	3.491	0.320	0.020	0.106	0.122

The “<“ sign means that the test result was obtained below the lower limit of the accreditation range, which does not constitute a result but only information about the content/concentration level of the tested factor. The numerical value after the < sign is the lower limit of the measurement range of the accredited method. Benzene, toluene, and ethylbenzene: method range: 0.0010–2.00 mg/sample. Xylene and total BTEX: scope of the method: from calculations.

**Figure 7:**
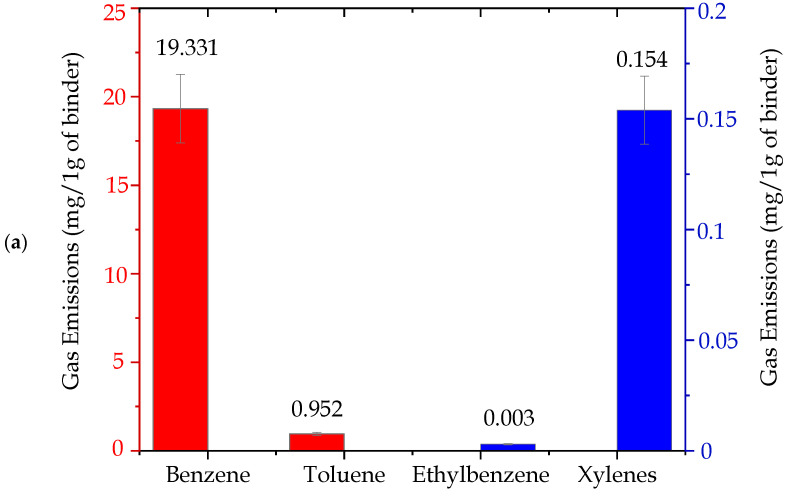

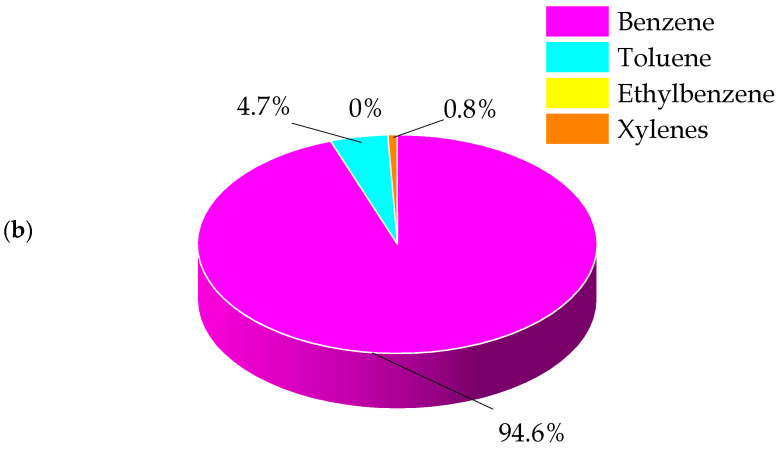
Contents of individual substances from the BTEX group emitted during pouring the molding sand (code no. 4) bonded by an organic binder: (**a**) per 1 g of a binder (red group (Benzene, Toluene)—scale on the left; blue group (Ethylbenzene, Xylenes)—scale on the right) and (**b**) percentage composition of gases emitted from the BTEX group.

**Figure 8:**
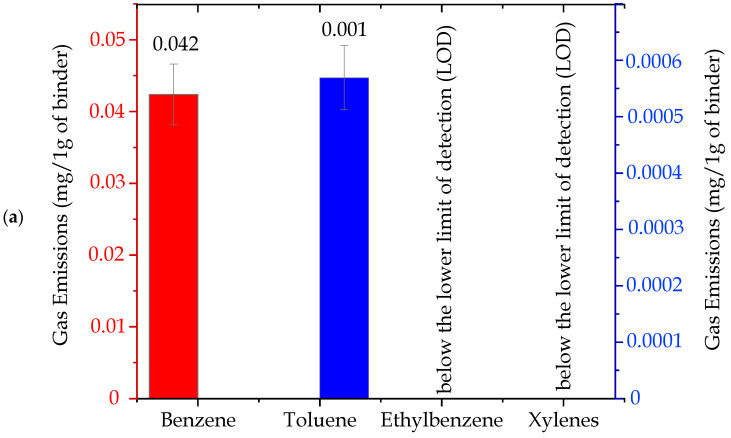

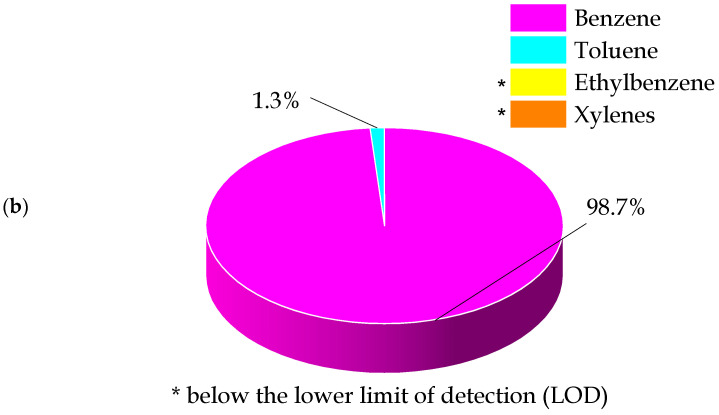
Contents of individual substances from the BTEX group emitted during pouring the molding sand (code no. 1) bonded by an inorganic binder: (**a**) per 1g of a binder (red group (Benzene, Toluene)—scale on the left; blue group (Ethylbenzene, Xylenes)—scale on the right) and (**b**) percentage composition of gases emitted from the BTEX group.

The main component of the released gases from the BTEX group, was—in all analyzed cases—benzene (Table 2). Its participation constituted >90% of the mixture of substances. Examples of investigation results of the emission of individual components from the BTEX group as well as their percentage participation in gases released from sands no. 4 and no. 1 are shown in Figure 7a,b and Figure 8a,b.

*Influence of the hardening technology on the emission of compounds from the BTEX group presented on the example of an inorganic binder: geopolymer or based on sodium silicate.*

Investigations of compounds from the BTEX group in gases emitted from molding sands have indicated that the applied hardening technology has a significant influence on the emission amount (Figure 6 and Table 2). In cases of molding sands with a geopolymer binder, the higher content of compounds from the BTEX group characterized sands hardened by CO_2_, while the same emission from sands hardened in warm box technology was approximately 20 times lower (Figure 6 and Table 2). Also, in cases of molding sands bonded by silicate binders, codes no. 5 and no. 7, the influence of a hardening factor is visible. In addition, in cases of sands hardened by CO_2_ (sand code no. 7) (∑ BTEX = 1.06 mg/1 g of a binder), the total emission of BTEX in the emitted gases was three times lower than in cases of sands hardened in the warm box technology (sand code no. 5, ∑ BTEX = 3.204 mg/1 g of a binder) (Figure 6 and Table 2).

*Influence of the protective coating on the total emission of substances from the BTEX group*. 

Performed investigations indicated that the presence of coating supports the formation of substances from the BTEX group (Figure 6 and Table 2). The total content of substances from the BTEX group emitted during pouring sands without protective coatings was lower than from sands with such coatings in cases of sand with an organic binder (code no. 4 + coating) as well as in cases of sand with an inorganic binder (code no. 3 + coating) (Figure 6 and Table 2).

#### 2.2.2. PAHs Emissions

**Figure 9:**
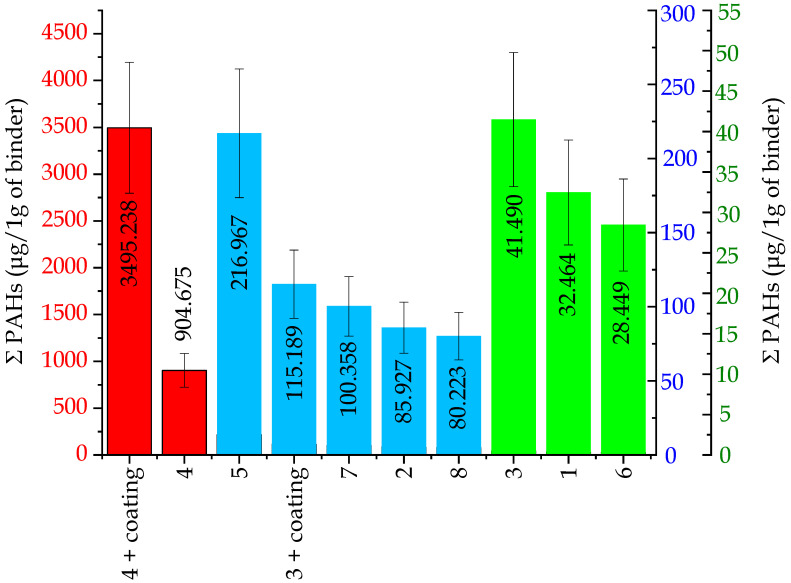
Total PAHs emission, calculated per 1 g of a binder (I red group (code: no. 4 + coating, no. 4)—very high content of substances from the PAHs group; II blue group (code: no. 5, 3 + coating, 7, 2, 8)—small content of substances from the PAHs group; III green group (code: no. 3, 1, 6)—trace content of substances from the PAHs group).

Investigation results of the emission of substances from the PAHs group, formed and released from tested molding sands during the casting production, are presented in Figure 9. In a similar way as in the case of testing the emissions of compounds from the BTEX group, in this case, three groups of molding sands differing in the contents of PAHs substances in emitted gases can be singled out. The first group, which contains molding sands of code no. 4 and code no. 4 + coating, emits PAHs substances at a level from 904.67 to 3459.23 µg/1 g of a binder, respectively. The second group (molding sands of code: no. 2, no. 3 + coating, no. 7, no. 8) is characterized by lower contents of these substances in gases: from 80.22 to 217.96 µg/1 g of binder. The third group (molding sands of code: no. 1, no. 3, and no. 6) has the lowest amount of PAHs substances in emitted gases (from 28.4 to 41.49 µg/1 g of a binder (Figure 9)).

The investigation results of the emissions of individual substances from the PAHs group are listed in Table 3. Eight of these substances (marked **) are classified as having probable carcinogenic and/or mutagenic activity. It is worth noting that naphthalene was the main component of the PAHs mixture in all of the tested cases (Table 3). In addition, the emission of benzo(a)pyrene was the highest for molding sands no. 4 and no. 4 + coating (bonded by an organic binder), while for molding sands bonded by inorganic binders, the emission was significantly lower (at least by one order of magnitude) (Table 3). 

*Influence of the hardening technology on the emission of substances from the PAHs group, presented on the example of inorganic binders: geopolymer- or sodium silicate-based*.

Investigations of the composition of emitted gases have indicated that the total contents of substances from the PAHs group depend on the hardening method (Figure 9 and Table 3). Above two times higher emissions of these substances were found in gases released by sands with a geopolymer binder hardened by CO_2_ than by sands hardened in the warm box technology, where the emission was only equal to 32.46 µg per 1 g of a binder (code no. 1) (Figure 9 and Table 3). However, in cases of sands based on sodium silicate hardened by CO_2,_ the total PAHs emission was two times lower (∑ PAHs = 100.358 µg/1 g of a binder) than in cases of sands hardened in the warm box technology (sand STX, ∑ PAHs = 216.967 µg/1 g of a binder (Figure 9 and Table 3).

*Influence of the protective coating on the total PAHs emission.*

The performed investigations indicate that the influence of coating on the PAHs substances emission was of a similar character as the one concerning substances from the BTEX group. The presence of the water-based protective coating type A created favorable conditions for forming as well as for releasing higher amounts of PAHs substances, as compared with sands without coatings (Figure 9 and Table 3).

Our previous studies [19,32,33] concerning investigations of the emissions of substance from the BTEX group indicated that in cases of sands with the participation of inorganic binders from the so-called “old generation”, marked as MG, MC, and MI, the obtained BTEX emission results were at the levels of 3.342, 0.715, and 0.860 mg/per 1 g of a binder, respectively. These results indicate that the presently tested inorganic binders of the new generation, especially the ones belonging to the III group (green group, no. 3, 6, 1) (see Figure 6 and Table 2), are characterized by significantly lower emissions of compounds from the BTEX group in comparison with binders of the old generation.

A similar tendency is observed in cases of emissions of compounds from the PAHs group. In cases of binders of the new generation, this emission was significantly lower (see Figure 9 and Table 4) in comparison with the emission results from the inorganic binders of the old generation, presented in papers [19,32,33]. As an example, for MC sand, the emission of PAHs was 66 µg/1 g of binder [19], whereas the emission from the binder of the new generation (no. 1) was two times lower and equaled 32.4 µg/1 g of binder (Figure 9 and Table 3). 

These results suggest that the direction of activities undertaken by the producers of binders, containing the production of binders of the new generation, is the proper one, and its continuation is justified on account of environmental protection and work conditions. 

#### 2.2.3. Emission of Other Harmful Organic Compounds

##### Phenol Emission

Measurements of the phenol content in gases released by molding sand samples indicated its presence in gases emitted only by molding sand code no. 4 (the reference sand bonded by an organic binder). It was equal to 0.055 mg per 1 g of a binder. In the remaining cases, the phenol content was below the detection limit of the applied method. 

##### Formaldehyde Emission

**Figure 10:**
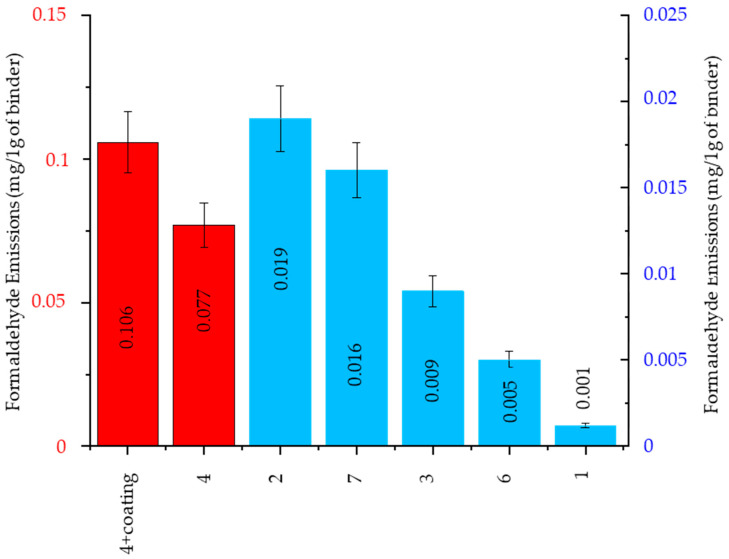
Formaldehyde emission, calculated per 1 g of a binder (red group (code: no. 4 + coating, no. 4)—the highest formaldehyde emission among those tested; blue group (code: no. 2, 7, 3, 6, 1)—trace emission of formaldehyde among those tested).

The investigation results of the formaldehyde content in gases emitted during molding sands and pouring with liquid alloys are presented in Figure 10. Tests showed that the formaldehyde presence in gases was emitted from the reference molding sand bonded by an organic binder (code no. 4 and no. 4 + coating). In addition, the formaldehyde presence was also found in gases released from molding sands bonded by inorganic binders (Figure 10), but in this case, the emission values were much smaller than in cases of the reference molding sand (code no. 4 and also code no. 4 + coating). It is worth noting that in cases of sand code no. 1, the emission value was just a trace (Figure 10). The presence of formaldehyde in gases emitted from sands bonded by inorganic binders is probably the effect of the thermal decomposition of organic additions applied to these sands.

##### Isocyanates Emission ∑ MDI/TDI

**Figure 11:**
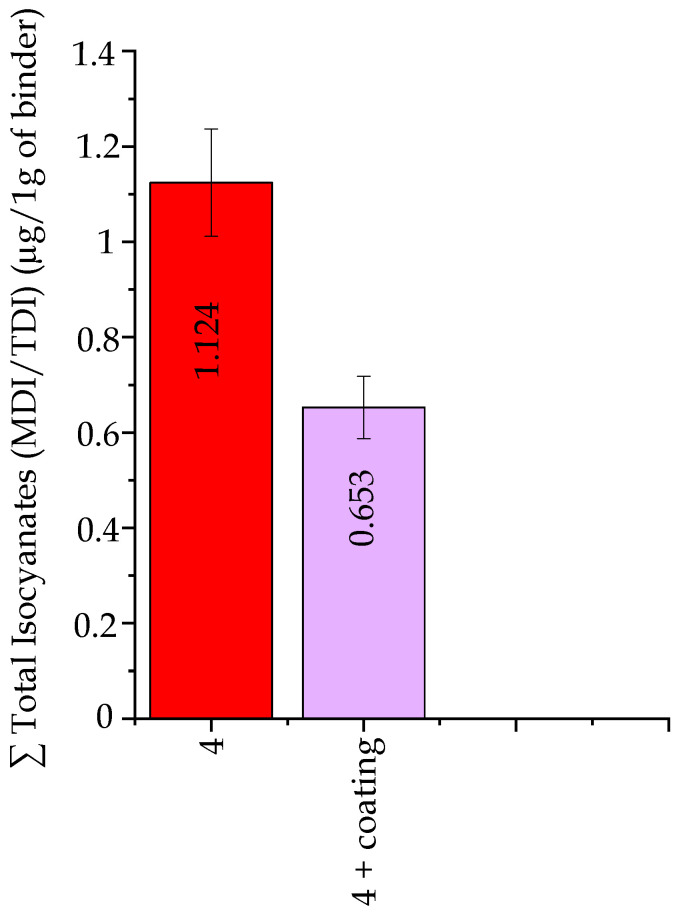
Total isocyanates (MDI/TDI) emission, calculated per 1 g of molding sand and per 1 g of a binder.

Investigations of the emissions of compounds from the isocyanates (MDI/TDI) group in gases released by molding sands of code no. 4 and no. 4 +coating indicated that the coating lowers the presence of isocyanates by nearly 50%, causing a decrease from 1.12 µg per 1 g of a binder (code no. 4) to 0.65 µg per 1 g of a binder (code no. 4 + coating) (Figure 11).

#### 2.2.4. Greenhouse Gases CO, CO_2_, NO_x_, SO_2,_ and T_voc_ Emissions

**Figure 12:**
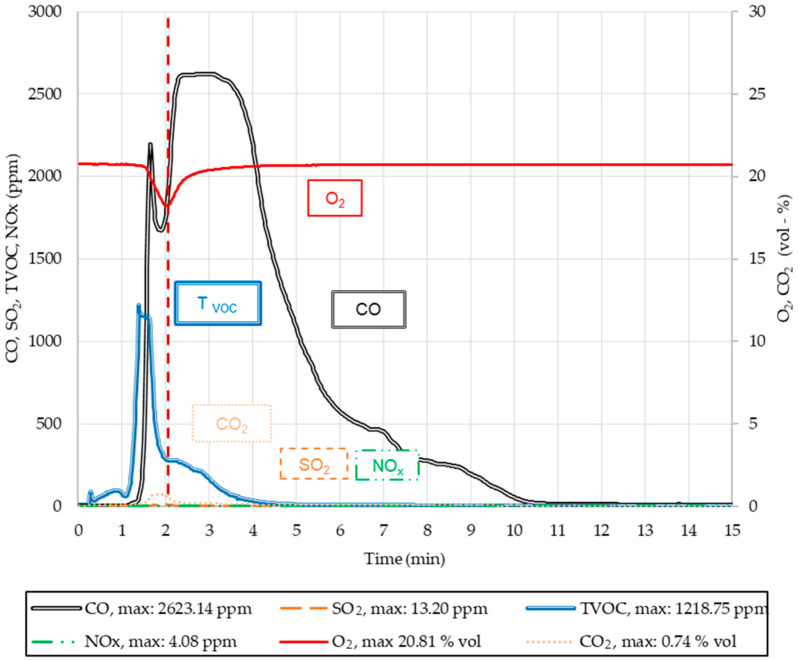
Concentration changes in CO, CO_2_, NO_x_, O_2,_ and T_voc_ in gases released from the molding sand code no. 1 (red dashed line—minimum O_2_ concentration in emitted gases).

**Figure 13:**
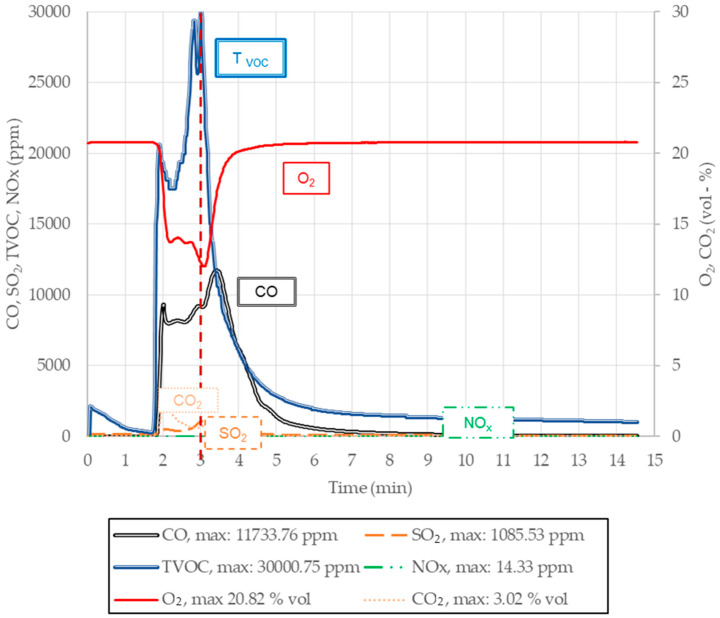
Concentration changes in CO, CO_2_, NO_x_, SO_2,_ and T_voc_ in gases released from the investigated molding sand (code no. 4) (red dashed line—minimum O_2_ concentration in emitted gases).

**Figure 14:**
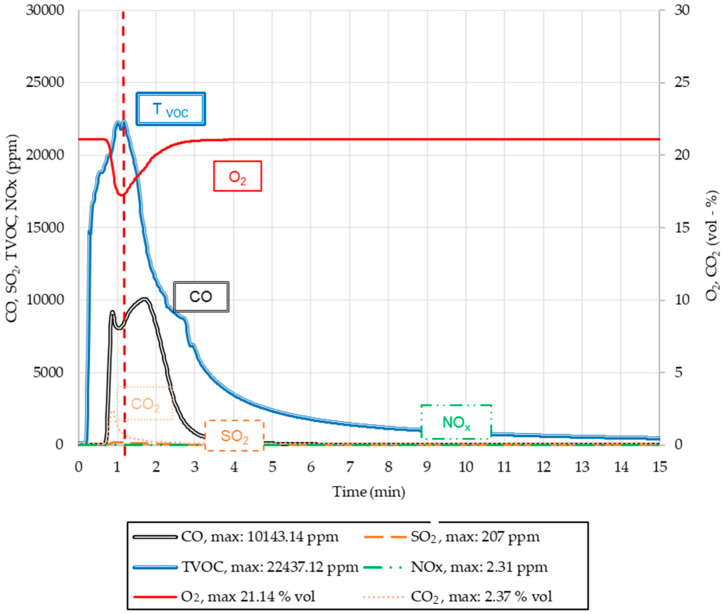
Concentration changes in CO, CO_2_, NO_x_, SO_2,_ and T_voc_ in gases released from the investigated molding sand (code no. 4 + coating) (red dashed line—minimum O_2_ concentration in emitted gases).

The results of gas volumetric emissions of the tested molding sands, from the point of view of liberating CO, CO_2_, NO_x_, SO_2,_ and T_voc_ during the pouring, are listed in Table 4. Visual pathways of the releasing profiles of individual substances are presented in Figure 12, Figure 13 and Figure 14.

**Table 4:** Maximal values of CO, CO_2_, NO_x_, SO_2,_ and T_voc_ emissions.

Molding Sand Code	Parameters											
	O_2_ (vol %)		CO (ppm)		CO_2_ (vol %)		NO_x_ (ppm)		SO_2_ (ppm)		T_voc_ (ppm)	
	^!^Max Value	^$^t_max_ (min)	^!^Max Value	^$^t_max_ (min)	^!^Max Value	^$^t_max_ (min)	^!^Max Value	^$^t_max_ (min)	^!^Max Value	^$^t_max_ (min)	^!^Max Value	^$^t_max_ (min)
1	20.80	3.0	2620	3.0	0.74	1.8	4.08	2.0	13.2	2.0	1220	1.3
2	20.80	2.2	2540	1.85	5.19	1.0	8.63	1.0	132	1.0	10,200	0.8
3	20.90	5.0	11,800	2.5	3.98	1.5	11.80	1.7	280	1.6	14,400	1.3
4	20.80	5.0	11,700	3.5	3.02	2.0	14.30	3.0	1080	3.0	30,000	0.6
4 + coating	21.10	3.0	10,100	1.8	2.37	0.8	2.30	1.1	207	1.1	22,400	1.2
5	20.90	4.0	2610	3.2	1.4	1.2	9.71	0.8	205	0.8	11,300	0.5
6	20.80	4.0	2660	4.0	3.61	1.0	7.16	1.1	169	1.1	8880	0.5
7	20.70	3.0	2560	2.1	2.90	1.5	8.57	1.8	75.7	1.8	9000	1.0
8	21.10	2.2	10,000	3.0	1.82	1.1	1.35	1.1	171	1.1	21,900	0.6

^!^Max value—maximal value of the concentration of the given gas in a mixture. ^$^t_max_—time from the moment of pouring, after which the emission of gases was at its maximum.

It can be noted that, in the graph below, presented as an example (Figure 12), after approximately 1 min of pouring, an intensive increase in releasing T_voc_ is seen, and then CO occurs from the sample of sand no. 1. The emission of CO continues for nearly 10 min after pouring, and it obtains very high values (CO_max_ = 2620 ppm). Gases such as CO_2_, SO_2,_ and NO_x_ (Figure 12 and Table 4) are—in practice—not released from molding sands during the pouring process. In addition, a small amount of oxygen is observed for approximately 2 min.

It can be observed that in the case of molding sand no. 2, approximately 30 s after pouring the mold, organic substances (T_voc_) oxidize and CO and CO_2_ are formed, which causes a decrease in the oxygen content (Table 4). The emissions of SO_2_ and NO_x_ are, in practice, equal to zero (Table 4). In cases of molding sand no. 3, already 1 min after pouring the mold, a violent increase in the amounts of T_voc_ and CO and a slow increase in CO_2_ occur in the emitted gases. After reaching the maximum value, the concentration of CO gradually decreases for approximately 6 min. The emissivity of NO_x_ and SO_2_ is at a trace level (Table 4). On the other hand, from the reference molding sand no. 4 (Figure 13), approximately 2 min after the moment when the mold was fully poured, a violent emission of gases such as T_voc_, CO, and CO_2_ started. The highest concentration of T_voc_ was noticed after approximately 3 min of pouring, followed by a sudden decrease in its emissivity, lasting approximately until the 5th minute. At the initial stage of pouring, trace amounts of CO_2_ and SO_2_ were found, while NO_x_ was not detected at all (Figure 13 and Table 4).

In cases of molding sand of code no. 4 + coating, presented in Figure 14, the release of T_voc_ gases is seen directly after pouring the mold. They obtain their emissivity maximum after 1 min, and it falls to zero after approximately 6 min. Approximately 30 s after pouring, CO and CO_2,_ also start emitting, reaching the emissivity maximum after approximately 1 min and 2 min, respectively. The emissivity of these gases ends after approximately 6 min. The presence of SO_2_ and NO_x_ in released gases was—in practice—not found (Figure 14 and Table 5).

Tests of molding sand no. 5 indicated that, directly after pouring the mold with a liquid casting alloy, violent emissions of T_voc_ and much less intense emissions of CO started. The emission of T_voc_ continues for approximately 5 min, while the emission of CO continues a little longer (for 8–9 min). NO_x_ and SO_2_ were not found, in practice, in released gases (Table 4). In the first minute after pouring, mold gases such as T_voc_, CO, and CO_2_ start emitting from molding sand no. 6. The release of CO continues for approximately 12 min, while the release of T_voc_ and CO_2_ ends after 3 min. In a similar fashion to the previously analyzed cases, the release of SO_2_ and NO_x_ is at a trace level (Table 4). Immediately after pouring, sand no. 7 substances from T_voc_ group were emitted. Their concentration violently increases, reaches a maximum in 1 min and then decreases to zero after 5–6 min. CO and CO_2_ start emitting after approximately 1 min after pouring sand no. 7, and their release continues to the third minute. The presence of SO_2_ and NO_x_ was not found (Table 4). In cases of molding sand no. 8, the emission of substances from T_voc_ was seen directly after pouring the mold. Their concentration was found to violently increase, obtaining the maximum in 1 min, and 5 min after the mold was poured, their emissivity fell practically to zero. The release of CO and CO_2_ started a little later and lasted for approximately 2 min. The presence of SO_2_ and NO_x_ was not found (Table 4).

#### 2.2.5. Emissions of Particulate Matter PM 2.5 and PM 10

Detailed data concerning investigations of grain size distribution of dusts liberated during pouring molds of the tested molding sands, with the special emphasis on the participation of dusts PM 2.5 and PM 10, are listed in Table 5.

**Table 5:** The results of investigations of grain size distribution of dusts generated during pouring molds of the tested molding sands.

Molding Sand Code	PM 2.5 ^&^ % Share	PM 10 ^&^ % Share	Total Weight of Dust (mg/1 g of a Binder)
1	19–49.6	70.8–100	0.484
2	60.7–62.8	79.1–91.0	0.703
3	30.5–68.9	79.2–99.7	0.246
3 + coating	20.4–46.5	53.9–99.5	0.160
4	21.6–30.5	65.6–89.6	7.511
4 + coating	5.8–43.7	14.2–100	5.137
5	20.4–76.3	53.9–100	1.105
6	28.4–44.6	77.8–99.6	0.448
7	11.6–40.3	70.6–100	0.681
8	2.7–57.6	2.9–100	0.169

^&^ % share, constitute the average equivalent volume of the given fraction. At the assumption that the density in the whole volume of the sample is the same, the percentage participation can be treated as the sand percentage participation.

**Figure 15:**
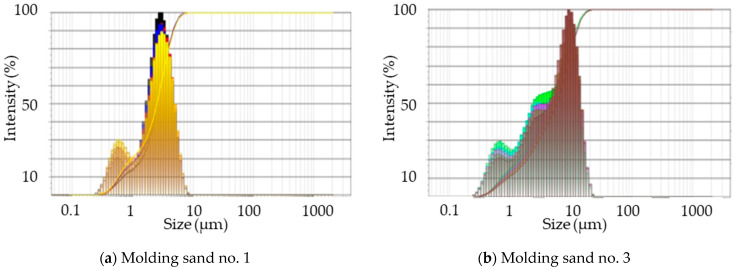

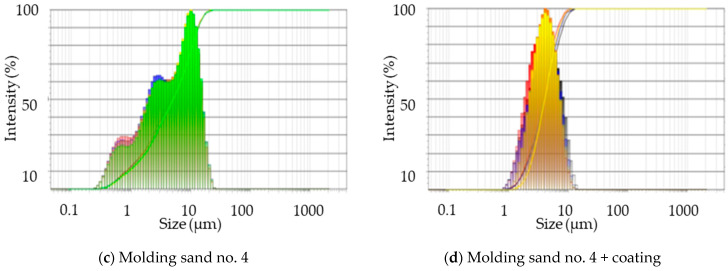
Examples of the results of the grain size distribution of dusts generated during pouring molds of the tested molding sands: (**a**) no. 1; (**b**) no. 3; (**c**) no. 4; and (**d**) no. 4 + coating.

In addition, the selected results of grain size distribution for molding sands of code no. 1, no. 3, no. 4, and no. 4 + coating are presented in Figure 15a–d. The largest amounts of dust were generated from samples with an organic binder (code no. 4). On average, it was 7.5 mg of dust calculated per 1 g of a binder (Table 5), while from sands with inorganic binders, the amount of generated dust was several times lower (the maximum result obtained for sample no. 5 was 1.1 mg per 1 g of a binder). On the basis of the performed research, it can be concluded that all of the 10 tested samples showed a high percentage of dust, PM 2.5 and PM 10. There were also samples with 100% PM 10 dust (Table 5).

## 3. Materials and Methods

### 3.1. Research Materials

The current emissions were measured in a reference system containing an organic binder and in new, tailor-made inorganic binder systems covered by the ongoing Green Casting Life project [31]. The subject of the research constituted molding sands based on 100% fresh sand and a specific amount of a binder, representing seven new, commercially available, inorganic binding systems and one organic binding system (see Table 6). These binders were not tested previously, but they are important for the production of iron castings in terms of meeting the environmental requirements.

In order to check the influence of the protective coating on the composition and amount of released gases, two molding sands were additionally selected for testing: one bonded with an organic binder, and one bonded with an inorganic binder. Both of them were covered with a water-based coating, type A.

The tested molding sand with the use of an organic binder code no. 4 reference has a much lower content of harmful monomers (especially phenol) than the classic binder from PUNB technology, thanks to which it is possible to significantly reduce emissions and improve the recovered sand quality. The designations of individual sands are given in Table 6. In these experiments, the test sand samples had the shape and dimensions of a typical cylindrical sample with a weight of 150 g and dimensions φ 50 mm × 50 mm. The temperature of liquid cast iron was 1380–1420 °C. The research was carried out in the experimental foundry at the Faculty of Foundry Engineering of the AGH University of Krakow, according to its own methodology described in several publications [5,19,20,34]. A detailed description of the methodology used, together with the chemical analysis, is presented in the authors’ previous works [5,19,20,35].

### 3.2. Methods

#### 3.2.1. Collection of Gas Samples for BTEX, PAHs, Phenol, Formaldehyde, and MDI/TDI Analysis

Measurements of the gas volume and BTEX, PAHs, phenol, formaldehyde, and MDI/TDI emissions in the small-scale test were performed according to the original method developed in the AGH University of Krakow (Poland), described in patent no. PL 224705 B1, 2017 [34]. Gases emitted from the sample—after its pouring with liquid metal—were directed into the pump by means of a steel pipe via the heating system, drying system, and the following:

- Sorption tubes (during the BTEX, phenol, or formaldehyde measurements).
- Polyurethane foam (PUF) (during the PAHs measurement).
- Special quartz filters (during MDI/TDI measurements).

The possibility of condensation of gaseous products in the measurement tubes and/or in capsules was eliminated by heating the gases transfer line to 100 °C. This heating was performed by the installation of the electrical resistance system in the housing, powered by its own DC source [19]. Adsorbed gases from the BTEX group, PAHs, phenol, formaldehyde, and MDI/TDI were extracted and prepared for chemical analysis, including chromatographic and spectroscopic techniques. 

#### 3.2.2. Collection of Gas Samples for CO, CO_2_, NO_x_, SO_2,_ and T_voc_ Analysis

The concentrations of greenhouse gases were measured online, directly during the casting process. The volume concentration of oxygen (O_2_) present in waste gases was determined using the HORIBA PG-350EU P-AMS gas analyzer (Horiba, Kyoto, Japan) operating on the basis of the Paramagnetic Method (PMD, PN-EN 14789:2017-04 [36]). The concentration of nitrogen oxides (NO_x_) was measured using the Chemiluminescent Method (CLD, PN-EN 14792:2017-04 [37]) with the application of the HORIBA PG-350EU P-AMS gas analyzer. The concentrations of carbon oxide CO (PN-EN 15058:2017-04 [38]), carbon dioxide CO_2_ (PN-ISO 10396:2001 [39]), and sulfur dioxide SO_2_ (PN-ISO 10396:2001 [39]) were measured using the HORIBA PG-350EU P-AMS gas analyzer with the application of the Non-Dispersive Infrared (NDIR) spectroscopy method.

The mass concentration of total volatile organic compounds (T_VOC_) was measured—using the flame ionization detection (FID) method—by the JUM OVF–3000 analyzer (JUM Engineering, Karlsfeld, Germany), which operates on the basis of the FID method (PN-EN 12619:2013-05 [40]). This method involves the ionization of carbon atoms present in organic compounds during the combustion of a mixture of waste gases, hydrogen, and pure air in a burner. All elements in contact with the sample were heated to a temperature of 180 °C. This prevents the loss of hydrocarbons and ensures the accuracy of the results. The analyzer has an internal control system that ensures the pressure control and stability of the sample flow through the combustion chamber. The flows of fuel and air are controlled using precision needle valves.

#### 3.2.3. Collection of Dust Samples for Determination of Particulate Matter PM 2.5 and PM 10

An analysis of the grain size of dust [41] generated by the tested molding sands and released during pouring the molds was carried out by means of the laser diffraction method using the ANALYSETTE NanoTech laser (FRITSCH, Weimar, Germany) particle meter in the range of 0.01–2100 µm. An effective analysis of the grain size distribution of the collected dust, with a particular emphasis on the distribution of PM 2.5 and PM 10 particles, was carried out using the test procedure, according to ISO 13320:2009 [42] and ISO 14488:2007 [43]. The measuring cell contained sample particles prepared for dispersion by the unit and illuminated by a laser beam. By changing the distance between the detector and measuring cell, a different angular range of scattered light was captured each time. The grain size distribution was determined from the measurement data.

## 4. Conclusions

The investigated systems of the inorganic binders group in the form of a new generation binders were not so far the subject of such tests. This means that the obtained new results of the composition of emitted gases will constitute a good supplementation of the former studies presented in the literature, which concentrated mainly on organic binders. These types of data, determining comprehensively several compounds from the PAHs, BTEX, phenol, and formaldehyde, dusts PM 2.5 or PM 10, obligatory in the European Union in relation to the air quality, are unique in relation to investigations of the harmfulness of binders used in foundry practice. 

The results of the comparison of the seven inorganic binders (code no. 1–3 and code no. 5–8) presented in this article with the organic binder based system (code no. 4), currently in use, which belongs to the PUNB no-bake group, clearly indicate that the use of systems based on inorganic binders will have a beneficial effect on the environment and working conditions. This applies, in particular, to parameters such as the volume of released gases and their composition, with particular emphasis on the content of substances from the BTEX, PAHs, phenol, formaldehyde, MDI/TDI, CO, CO_2_, NO_x_, SO_2_, and Tvoc groups, as well as dust (PM 10, PM 2.5) released from molding sands when these sands are poured with liquid casting alloys. The use of an inorganic binder in molding sand, e.g., code no. 1, in comparison with sand with an organic binder (code no. 4 + coating), results in a nearly 100% (99.8%) reduction in emissions of substances from the BTEX group, and in cases of the use of molding sand of code no. 7, a reduction of 90.8% is achieved. Similar levels of reduction (around 90%) in the presence of harmful substances in the emitted gases were obtained for the remaining sands bound with inorganic binders. 

In an effort to improve the environment and working conditions, it is very important to change the approach to using less harmful materials as binding additives. In addition to environmental aspects, these additives must also meet technological requirements. It should be taken into account that the various additives to sands with inorganic binders (based on geopolymer substances or on modified sodium silicate) can considerably increase their low harmfulness since these additives introduce components that are a source of harmful emissions (BTEX, PAHs, phenol, formaldehyde, etc.), especially during the mold pouring stage. This was confirmed by the results of tests on some sands bound with inorganic binders, to which small additions of organic compounds were applied as catalysts or modifiers. 

However, in this case, the presence of harmful substances was several times lower than in the reference molding sand (code no. 4). The overall summary of the research indicates that the stated aim of reducing the negative impact of the foundry industry on work places and improving the environment requires the replacement of existing molding and core sand production technologies with organic-type binders with technologies adapted to the production of sands based on inorganic binders.

## Figures and Tables

**Table 1:** Average volume of gases emitted from 1 g of a binder (after 900 s of pouring).

Molding Sand Code	^ V_900 s_ (cm^3^/1 g Binder)	Max. Gas Release Rate (cm^3^/s) (per 1 g of Binder)	^^ T_max._ (s)
1	192.41	1.55	50–95
2	543.01	5.59	60
3	508.51	5.71	60
3 + coating	375.16	5.96	35
4	855.16	9.74	55
4 + coating	855.83	10.05	65
5	405.55	4.75	80
6	537.07	7.78	55
7	583.97	6.42	55
8	341.70	3.70	10

^ V_900_—volume of gases released from the tested sand samples during pouring (after 900 s from the mold being poured). ^^ T_max_—time after which (from the moment of pouring) the emissivity of gases was at its maximum rate.

**Table 3:** Contents of substances from the PAHs group generated by the thermal decomposition of molding sands components due to the influence of high temperatures of liquid casting alloys (during the production of castings).

PAHs	Emission of Individual Substances (µg/1 g of Binder)									
	Molding Sand Code									
	1	2	3	3 + Coating	4	4 + Coating	5	6	7	8
Naphthalene	21.541	63.878	22.465	93.307	375.127	1866.723	121.096	19.251	67.072	70.438
Acenaphthene	0.204	0.328	0.459	0.631	0.918	9.344	1.416	0.318	0.273	0.303
Fluorene	0.250	0.564	0.801	1.879	9.988	50.097	0.907	0.375	0.463	0.499
Phenanthrene	1.979	3.271	3.195	4.428	56.612	571.015	1.458	1.398	3.246	2.655
Anthracene	5.354	3.555	3.678	6.209	29.453	175.056	8.804	4.103	4.359	2.630
Fluoranthene	0.445	2.103	0.243	<	131.342	249.146	1.735	0.266	2.396	<
Pyrene	0.339	3.371	0.304	<	106.345	191.701	4.213	0.265	3.343	0.683
** Benzo[a]anthracene	0.057	0.617	1.233	0.219	16.296	61.852	0.833	0.048	1.697	0.068
** Chrysene	0.767	0.403	7.230	0.459	7.692	63.392	0.911	0.584	7.631	0.903
** Benzo[b]fluoranthene	0.096	1.322	0.109	1.102	30.961	64.230	7.439	0.082	1.754	0.204
** Benzo[k]fluoranthene	0.016	0.531	0.030	0.339	12.055	28.627	8.927	0.021	0.594	0.053
** Benzo(a)pyrene	1.054	1.500	1.029	1.819	34.132	64.511	30.129	1.514	2.180	0.838
** Dibenz[a,h]anthracene	0.031	0.763	0.075	0.477	18.577	24.514	2.083	0.031	0.921	0.094
** Benzo[g,h,i]perylene	0.249	2.159	0.480	2.707	40.888	34.531	23.433	0.141	2.542	0.580
** Indeno [1,2,3c,d]pyrene	0.084	1.562	0.159	1.611	34.289	40.499	3.583	0.049	1.886	0.275
Σ PAHs	32.464	85.927	41.490	115.189	904.675	3495.238	216.967	28.449	100.358	80.223
the value of +/− 20% on account	6.493	17.185	8.298	23.038	180.935	699.048	43.393	5.690	20.072	16.045

** Considered probable or possible human carcinogenic by the U.S. Environmental Protection Agency (EPA), the European Union, and/or the International Agency for Research on Cancer (IARC); The “<” sign means that the test result was obtained below the limit of the accreditation scope, which is not the result but only the information about the level of content/concentration of the tested factor. The numerical value after the < sign is the lower limit of the measurement range of the accredited method. PAHs range methods: 0.050–5.0 µg per sample.

**Table 6:** Types of investigated molding sands and their codes.

Molding Sand Code	Technology of Molding Sand	Type of Hardened Agent	Type of Protective Coating
1	Mold sand with inorganic binder (geopolymer)	warm box technology	-
2	Mold sand with inorganic binder (geopolymer)	CO_2_	-
3	Mold sand with inorganic binder (silicate)	ester (no-bake technology)	-
3 + coating			water-based coating type A
* 4	Mold sand with organic binder	amine (PUNB no-bake technology)	-
* 4 + coating			water-based coating type A
5	Mold sand with inorganic binder (silicate)	warm box technology	-
6	Mold sand with inorganic binder (silicate)	ester (no-bake technology)	-
7	Mold sand with inorganic binder (silicate)	CO_2_	-
8	Mold sand with inorganic binder (silicate)	ester (no-bake technology)	-

* molding sand code 4—molding sand bonded with an organic binder, reference in emissions measurements.

## Data Availability

Data will be made available on request.
